# Quantitative evaluation of the infrapatellar fat pad in knee osteoarthritis: MRI-based radiomic signature

**DOI:** 10.1186/s12891-023-06433-7

**Published:** 2023-04-25

**Authors:** Qin Ye, Dong He, Xiaonan Ding, Yajie Wang, Yuguo Wei, Jing Liu

**Affiliations:** 1 Center for Rehabilitation Medicine, Department of Radiology, Zhejiang Provincial People’s Hospital, Affiliated People’s Hospital, Hangzhou Medical College, Hangzhou, Zhejiang China; 2 Precision Health Institution, General Electric Healthcare, Hangzhou, China

**Keywords:** Knee osteoarthritis, Radiomics, Infrapatellar fat pad, Magnetic resonance imaging

## Abstract

**Background:**

The infrapatellar fat pad (IFP) may have bilateral influence on knee osteoarthritis (KOA). IFP evaluation may be a key contributor to the diagnostic and clinical management of KOA. Few studies have evaluated KOA-related IFP alteration with radiomics. We investigated radiomic signature for the assessment of IFP for KOA progression in older adults.

**Methods:**

A total of 164 knees were enrolled and grouped based on Kellgren-Lawrence (KL) scoring. MRI-based radiomic features were calculated from IFP segmentation. The radiomic signature was developed using the most predictive subset of features and the machine-learning algorithm with minimum relative standard deviation. KOA severity and structure abnormality were assessed using a modified whole-organ magnetic resonance imaging score (WORMS). The performance of the radiomic signature was evaluated and the correlation with WORMS assessments was analyzed.

**Results:**

The area under the curve of the radiomic signature for diagnosing KOA was 0.83 and 0.78 in the training and test datasets, respectively. Rad-scores were 0.41 and 2.01 for the training dataset in the groups with and without KOA (P < 0.001) and 0.63 and 2.31 for the test dataset (P = 0.005), respectively. WORMS significantly and positively correlated with rad-scores.

**Conclusions:**

The radiomic signature may be a reliable biomarker to detect IFP abnormality of KOA. Radiomic alterations in IFP were associated with severity and knee structural abnormalities of KOA in older adults.

**Supplementary Information:**

The online version contains supplementary material available at 10.1186/s12891-023-06433-7.

## Background

Knee osteoarthritis (KOA) is the most prevalent type of degenerative knee joint disease, with an incidence up to 25% in elderly people, and causes pain and disability [1, 2]. KOA is considered a whole knee joint disease rather than a disease from cartilage degradation alone. Several knee joint structures including the infrapatellar fat pad (IFP) are involved in KOA [3, 4]. The IFP is an intracapsular but extrasynovial structure and functions as an anatomo-functional unit in combination with the synovial membrane [5]. It can stabilize and protect the knee from mechanical damage [6]. IFP pathological manifestations in KOA patients are typically characterized by inflammatory infiltration and fibrosis accompanied with alterations of biomechanical properties [3, 7, 8]. How IFP is involved in the pathogenesis of KOA is partially but not completely clear. Possible mechanisms include the following: (1) the immune response of IFP is activated by cartilage-specific autoantigens; (2) subsequent local inflammation is induced by a variety of cytokines and adipokines; and (3) IFP interacts with cartilage, subchondral bone, and synovial membrane [3, 6, 9–13]. Therefore, the IFP plays an important role in KOA. Better understanding of this process may provide more information of the progression, diagnosis and clinical management of KOA.

Noninvasive evaluation of KOA-related IFP is performed mainly by magnetic resonance imaging (MRI) [2, 3]. Parameters of MR signal intensity, shape, and volume in the IFP have been considered as biomarkers for structural abnormalities and inflammation severity in KOA [14–17]. However, this evaluation method is subjective and time-consuming and has poor inter-observer and intra-observer variability. Perfusion parameters on contrast enhanced MRI can assess KOA-related IFP alterations with good variability. However, the side effects, ethical issues, and high cost limit its application [18, 19]. In contrast, radiomics noninvasively captures subtle alterations of the entire tissue and lesion through quantitative radiomic features within conventional standard-of-care medical images [20]. This technique has shown promise in the musculoskeletal system, such as in the cartilage and subchondral bone radiomic analysis in KOA patients [21–23]. However, it has not been applied in the evaluation of IFP.

The purpose of this study was to develop and evaluate the ability of a radiomic signature for detecting IFP abnormality in KOA. We also determined the relationship of IFP radiomics alteration with knee structural abnormalities.

## Methods

### Data sets

**Fig. 1:**
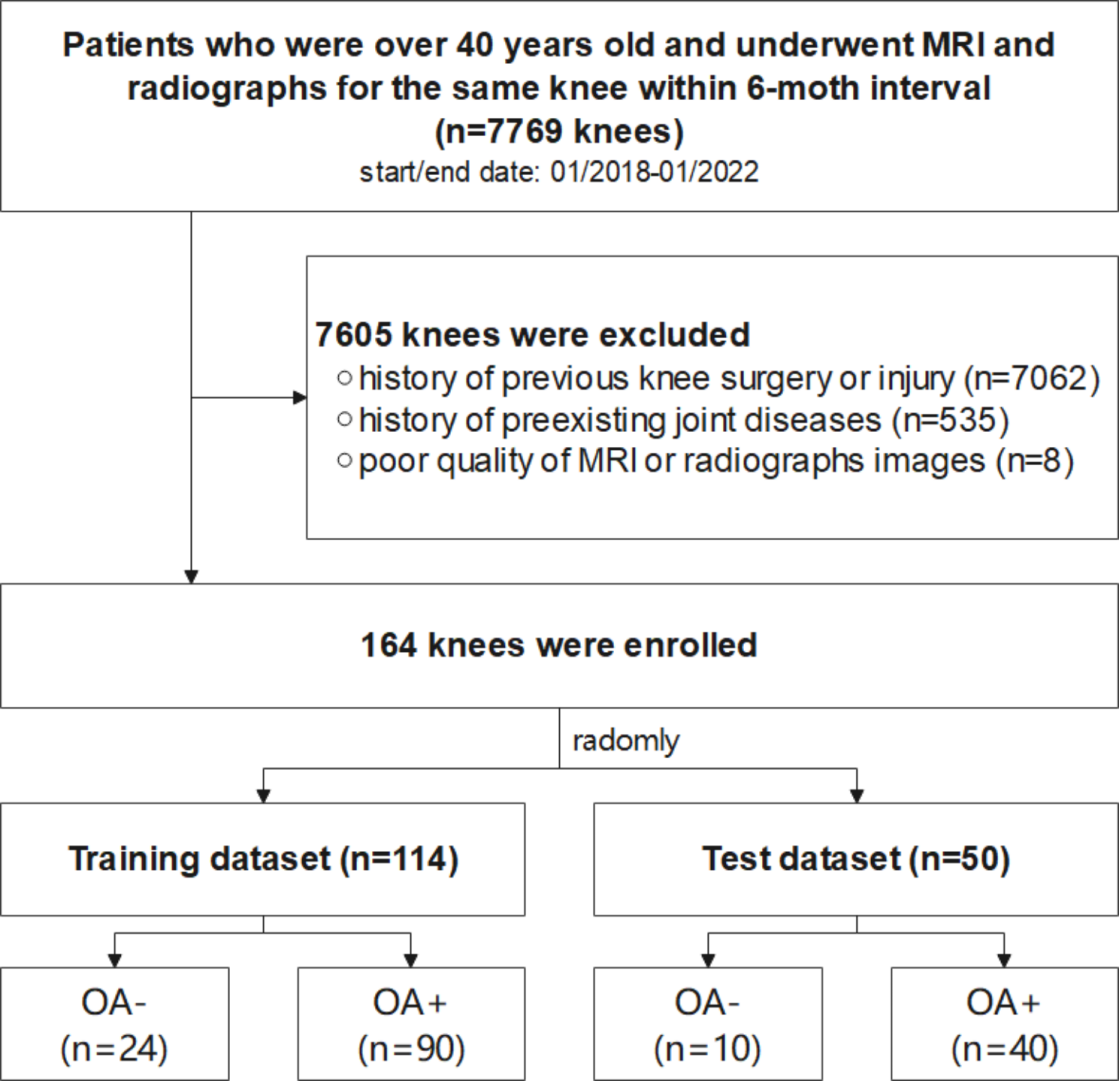
Flowchart of patient enrollment and dataset allocation. (OA−, knee without osteoarthritis; OA+, knee with osteoarthritis)

This retrospective study was approved by the institutional review board of ZJPP Hospital and informed consent was waived. All data were retrieved consecutively at our institution from January 2018 to January 2022 via the Picture Archiving and Communication System. Patients were eligible for inclusion if they were over 40 years old and underwent MRI and radiographs for the same knee within a 6-month interval. In case of multiple MRI and/or radiograph examinations for the same knees, paired examinations with the shortest interval were selected. The exclusion criteria were as follows: (1) history of previous knee surgery or injury; (2) history of preexisting joint diseases (e.g., inflammatory arthritis, osteonecrosis, metabolic disorder, or other diseases that affect IFP); and (3) poor quality of MRI or radiograph images. Finally, a total of 164 knees were enrolled in this study. The knees were randomly divided into the training dataset (n = 114) and test dataset (n = 50) at a ratio of 7:3 according to random numbers auto-generated by a computer (Fig. 1).

### Knee assessment

Knee assessments on radiographs and MRI were independently performed by two musculoskeletal radiologists (R1 and R2, with 13 and 18 years of experience, respectively), who were blinded to the clinical data. Disagreement in assessments were resolved by discussion among the two radiologists.

#### Plain radiographic assessment

The Kellgren–Lawrence (KL) scoring system [24] was used to score knee radiographs as follows: score 0, no change; score 1, doubtful osteophytes and joint space narrowing; score 2, definite osteophytes but suspicious joint space narrowing; score 3, moderate multiple osteophytes, definite joint space narrowing, some sclerosis, but possible bone deformity; and score 4, large osteophytes, marked joint space narrowing, severe sclerosis, and definite bone deformity. Using the KL scores [25], knees were classified into two groups: no detectable KOA with KL score of < 2 (OA^–^ group) and KOA with KL score of ≥ 2 (OA^+^ group).

#### MRI assessment

MRIs were performed using a 1.5T MRI scanner (Philips, Ingenia) with an 8-channel knee coil. Conventional T1-weighted and proton density-weighted fat-saturated images of the knee were obtained to evaluate the presence and grade of knee structural abnormalities (Supplementary Table S1). Cartilage, bone, meniscus, ligaments, and synovium of the joint structures were separately scored according to a modified whole organ magnetic resonance imaging score (WORMS) [26]. Cartilage defects were scored from 0 to 6, subarticular bone marrow abnormalities from 0 to 3, bone cysts from 0 to 3, bone attrition from 0 to 3, osteophytes from 0 to 7, meniscus from 0 to 6, ligaments from 0 to 1, effusion-synovitis from 0 to 3, and Hoffa-synovitis from 0 to 3 (Supplementary Table S2).

### Radiomics

Sagittal proton density-weighted fat-saturated images of the knee were used in the quantitative radiomics analyses, as follows:

#### Segmentation of IFP

**Fig. 2:**
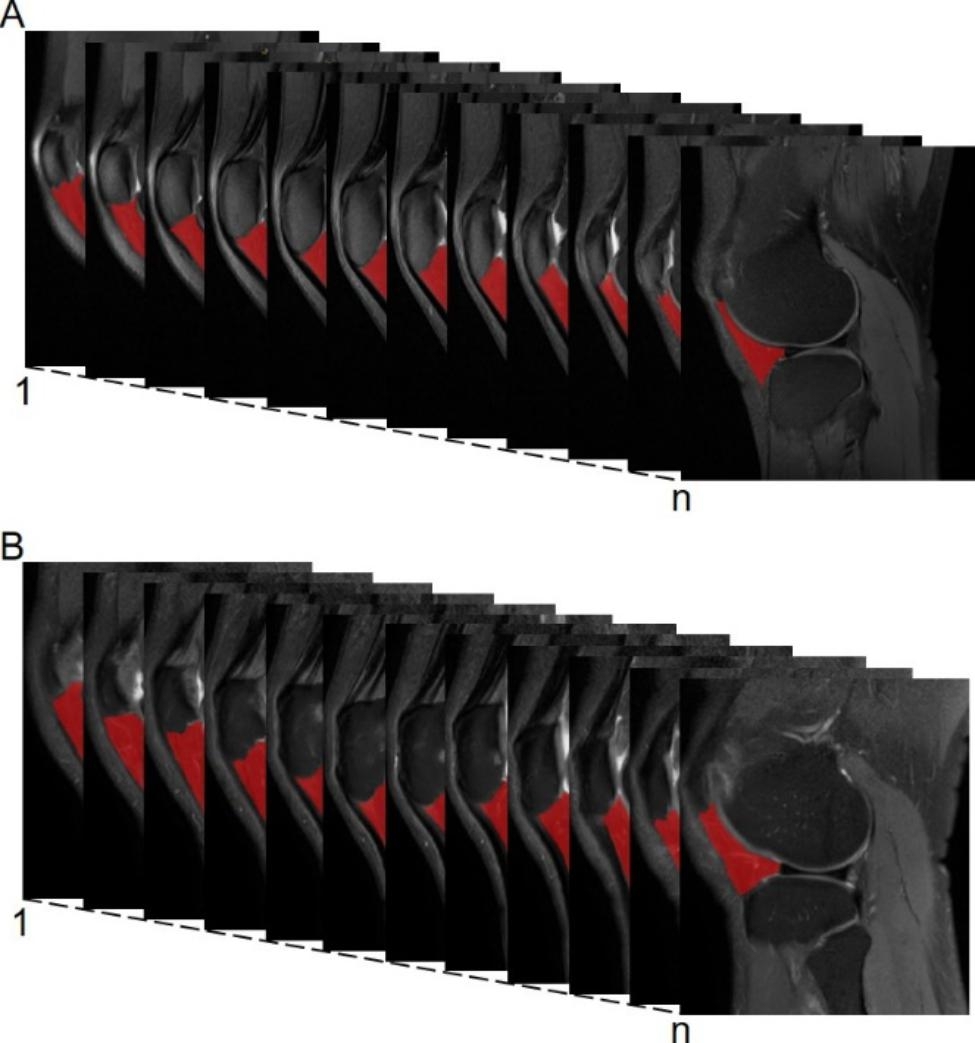
The VOIs of IFP for two representative cases from the OA − and OA + groups. (**A**) A 41-year-old woman with KL-1. (**B**) A 52-year-old man with KL-2. (VOI, volume of interest; OA−, knee without osteoarthritis; OA+, knee with osteoarthritis; KL, Kellgren–Lawrence).

The dedicated ITK-SNAP software (www.itksnap.org) was used for segmentation of IFP. Briefly, R1 drew a polygonal region of interest of the entire IFP territory slice-by-slice and then generated a volume of interest (VOI) for the subsequent feature extraction (Fig. 2). After a 6-week interval, 20 randomly selected images were independently segmented by R1 and R2, who were completely blinded to previous VOIs, for the inter- and intra-rater agreements.

#### Image preprocessing

Image preprocessing of images, including image resample, intensity normalization and gray-level discretization, was performed using PHIgo software (GE Healthcare, V1.5.0, China).

#### Feature extraction and selection

A total 1037 radiomic features were extracted from all VOIs via the PHIgo software including (1) shape features from original image and (2) first-order features, gray level cooccurrence matrix (GLCM) features, gray level size zone matrix (GLSZM) features, gray level run length matrix (GLRLM) features, neighboring gray tone difference matrix (NGTDM) features and gray level dependence matrix (GLDM) features from original, wavelet and Laplacian of Gaussian filtered images. A summary of the extracted features is shown in Supplementary Figure S1. The extracted features with an intraclass correlation coefficient (ICC) of > 0.75 were left because of stable parameters. Analysis of variance, minimum redundancy maximum relevance (mRMR), and Gradient Boosting Decision Tree (GBDT) was used for further feature selection by IPMs version 2.5.5.

#### Radiomic signature selection and development

First, the radiomic signature was developed on the basis of selected features by three machine-learning algorithms, including logistic regression (LR), Bayes, and support vector machine (SVM). The stability was quantified by the 1000 Bootstrap replication and its relative standard deviation (RSD), computed as the standard deviation divided by the corresponding mean value of the 1000 AUC values of each algorithm. Second, we selected the algorithm with minimal RSD for further analysis. Finally, the radiomics score (Rad-score) was calculated for each patient using the developed radiomic signature derived from the data in the training dataset. These procedures were performed using the IPMs software.

### Statistical analysis

Statistical analysis was performed using SPSS software (version 22.0) and R software (version 3.4.1). Statistical significance was defined at P < 0.05. Continuous data are reported as the mean ± standard deviation or median (interquartile range), and categorical data are reported as numbers (percentages). Normality testing was evaluated using the Shapiro–Wilk test. Differences in the general data distributions between training and test data were assessed using the two-sample t test, Mann–Whitney U test and chi-square test. Second, the inter- and intra-observer reliability of the IFP segmentation were assessed using ICC. The performance of the radiomic signature was evaluated by the area under the curve (AUC) of receiver operating characteristics (ROC) curve, accuracy, precision, sensitivity, and specificity; its goodness-of-fit was evaluated by the Hosmer–Lemeshow test. Finally, the association between WORMS and radiomic quantitative values was measured using Spearman’s rank correlation coefficient.

## Results

### General data characteristics

A total of 164 knees were included in this study and randomly classified into the training (n = 114) and test (n = 50) datasets. General characteristics including age, gender, side, body mass index (BMI) and Kellgren–Lawrence scoring in the datasets are summarized in Table 1. No significant differences were observed between the two datasets (*p* = 0.08–0.74).

**Table 1:** Characteristics of the training and test datasets

Characteristics	Training dataset (n = 114)			Test dataset (n = 50)			P_inter_ value
	OA-	OA+	P_intra_ value	OA-	OA+	P_intra_ value	
n	24	90	-	10	40	-	-
Age (year), median (IQR)	46.50 (44.50–55.00)	56.00 (50.00–70.00)	<0.01	47.50 (42.50–53.25)	59.00 (51.25–64.00)	<0.01	0.58
Gender, n (%)			0.63			0.56	0.08
Male	5 (20.8)	23 (25.6)		3 (30)	16 (40)		
Female	19 (79.2)	67 (74.4)		7 (70)	24 (60)		
Side, n (%)			<0.01			0.48	0.65
Right	7 (29.2)	57 (63.3)		5 (50)	25 (62.5)		
Left	17 (70.8)	33 (36.7)		5 (50)	15 (37.5)		
BMI (kg/m^2^), mean ± SD	22.36 ± 2.35	24.69 ± 2.46	<0.01	21.30 ± 1.43	25.11 ± 2.05	<0.01	0.74
KL-score, n (%)			<0.01			<0.01	0.58
KL-0	1 (4.2)	0		1 (10)	0		
KL-1	23 (95.8)	0		9 (90)	0		
KL-2	0	57 (63.4)		0	30 (75)		
KL-3	0	22 (24.4)		0	4 (10)		
KL-4	0	11 (12.2)		0	6 (15)		
Rad-score, median (IQR)	0.41 ( -0.34–1.11)	2.01 (1.47–2.70)	<0.01	0.63 (0.16–1.44)	2.31 (1.22–2.87)	<0.01	0.85

Note. OA-, knee without osteoarthritis; OA+, knee with osteoarthritis; IQR, interquartile range; n, number; KL, Kellgren-Lawrence; SD, standard deviation. P_intra_ value indicates significant differences between OA- and OA + groups in each dataset. P_inter_value indicates significant differences between the training and test datasets

### Intra- and inter-observer reliability

The inter-observer reliability on the extracted features between the two IFP segmentations from the two raters was greater than 0.79. The intra-observer reliability on the extracted features between the two IFP segmentations from one rater was greater than 0.82.

### Selection of the radiomic feature and machine-learning algorithm

#### Radiomic feature selection

**Fig. 3:**
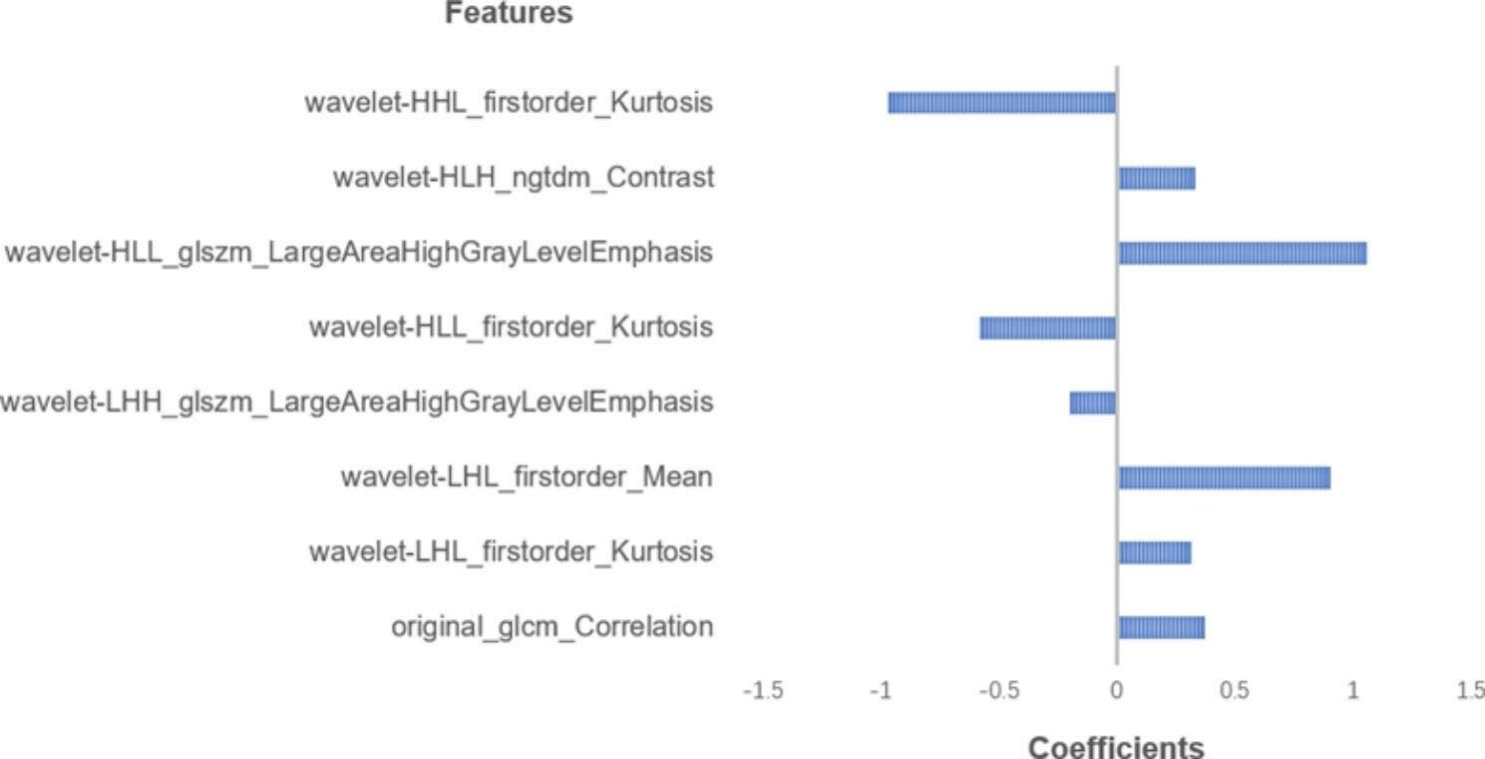
Selected features and the corresponding weight coefficients

The 419 and the 24 features were retained by analysis of variance and mRMR in turn. The eight most valuable features were identified by GBDT, including original_glcm_Correlation, wavelet-LHL_firstorder_Kurtosis, wavelet-LHL_firstorder_Mean, wavelet-LHH_glszm_LargeAreaHighGrayLevelEmphasis, wavelet-HLL_firstorder_Kurtosis, wavelet-HLL_glszm_LargeAreaHighGrayLevelEmphasis, wavelet-HLH_ngtdm_Contrast, and wavelet-HHL_firstorder_Kurtosis (Supplementary part I and Fig. 3).

#### Machine-learning algorithm selection

The RSD of LR (5.74%) was lower than those of Bayes (6.57%), and SVM (18.53%) (Supplementary part II). Therefore, the LR was chosen to construct the radiomic signature, with a linear combination of the selected eight features weighted by their corresponding weight coefficients (Fig. 3).

### Development and evaluation of radiomic signature

**Fig. 4:**
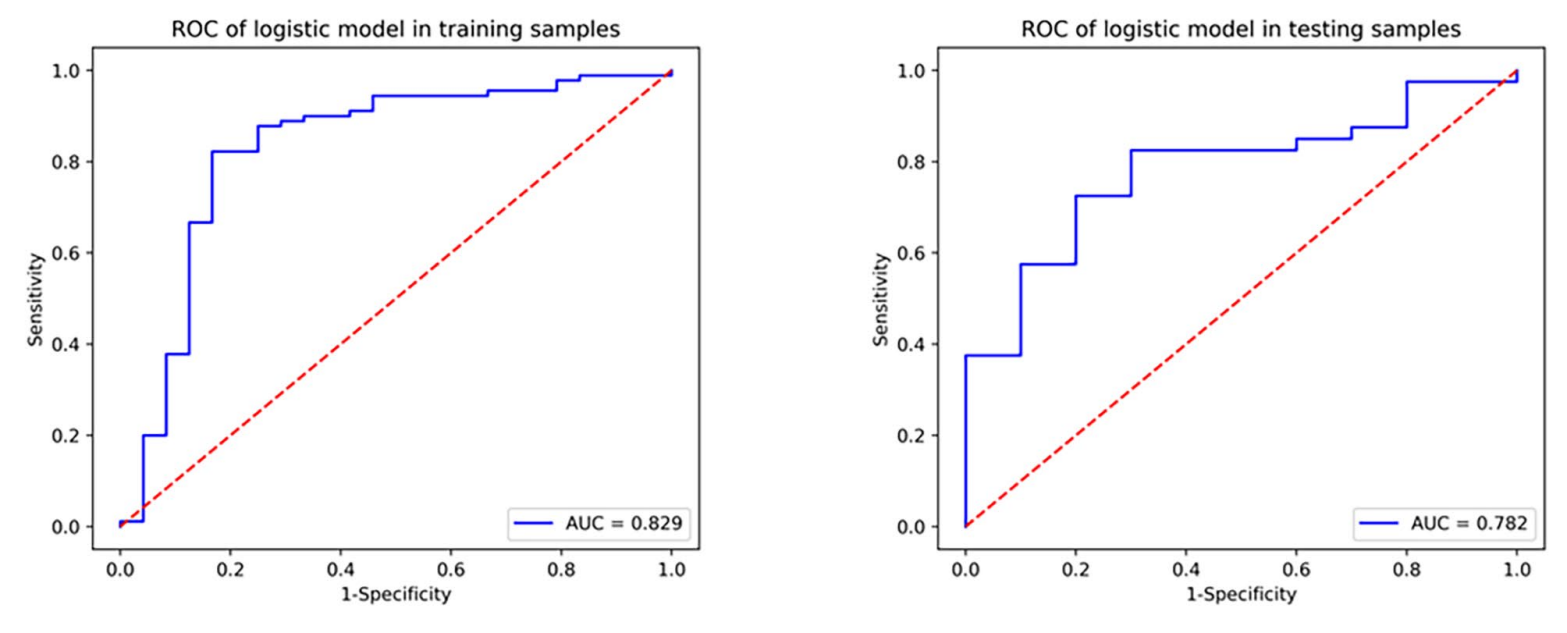
The receiver operating characteristic curve of the IFP radiomic signature for predicting KOA in the training (**A**) and test datasets (**B**)

The formula of rad-score based on LR and the eight selected features is shown Supplementary part III and was calculated for each patient. The radiomic signature showed good predictive performance for KOA in the training and test datasets (Table 2; Fig. 4). In the training dataset, the AUC, accuracy, precision, sensitivity, and specificity of the radiomic signature were 0.83 ((95% confidence interval [CI]: 0.74–0.92), 81.6%, 94.8%, 81.1%, and 83.3%, respectively. The Hosmer–Lemeshow test revealed no overfitting (*p* = 0.46). In the test dataset, the AUC, accuracy, precision, sensitivity, and specificity of the radiomic signature were 0.78 (95% CI: 0.65–0.90), 72%, 90.6%, 72.5%, and 70%, respectively.

**Table 2:** The predictive performance of radiomic signature in the training and test datasets

	AUC (95%CI)	Accuracy (%)	Precision (%)	Sensitivity (%)	Specificity (%)
Training dataset	0.83 (0.74–0.92)	81.60	94.80	81.10	83.30
Test dataset	0.78 (0.65–0.90)	72.00	90.60	72.50	70.00

Note. AUC, area under the curve, CI, confidence interval

### Differences of radiomic signature between the two groups

**Fig. 5:**
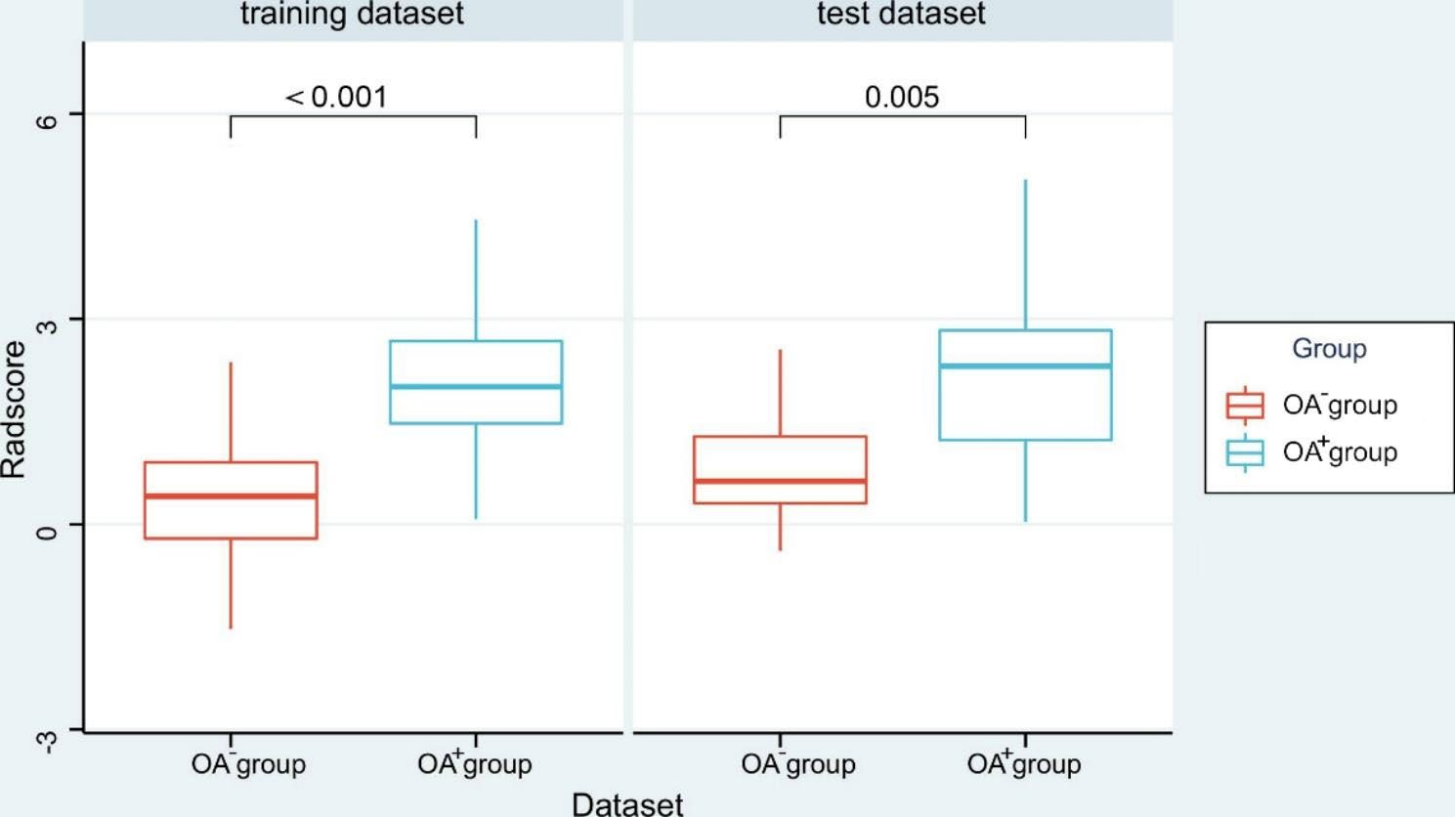
Summary of radiomic signature statistics between the OA − and OA + groups in the training and test datasets

In the training and test datasets, both of the IFP rad-scores were lower in the OA^–^ group (0.41–0.63) compared with OA^+^ group (2.01–2.31), with significant differences between the two groups (P < 0.001 and P = 0.005, respectively). No significant difference was observed between the two datasets (P = 0.85) (Table 1; Fig. 5).

### Correlations between radiomic signature and WORMS

**Fig. 6:**
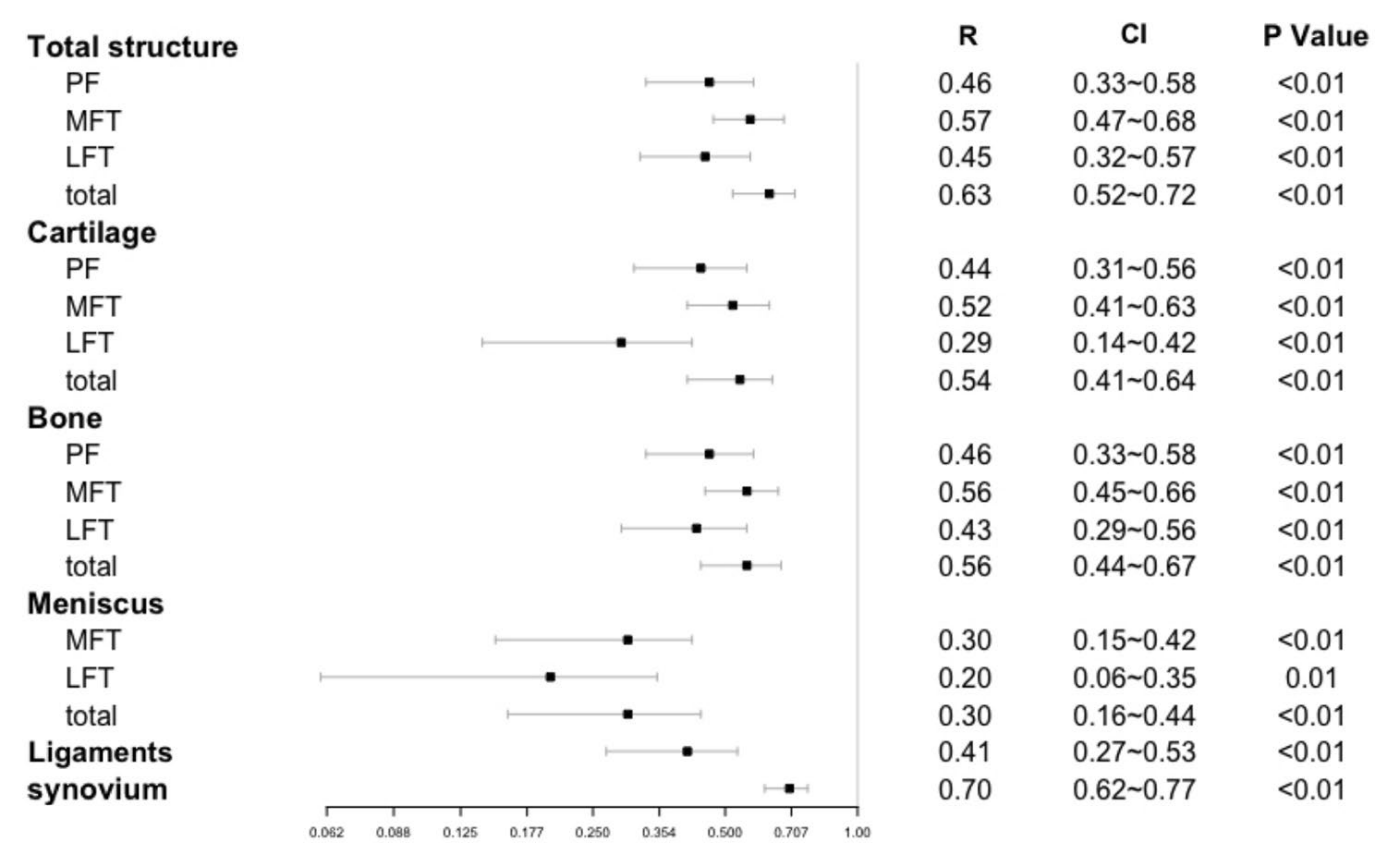
Forest plot for the correlations between radiomic signature and WORMS. “Total” (in bold) represents all structures of cartilage, bone, meniscus, ligaments, and synovium; “Total” (no bold) represents all regions of PF, MFT, and LFT.

Significant and positive correlations were found between rad-scores and WORMS scores of total knee structures (r = 0.45–0.63, all *p* < 0.01). Significant and positive correlations were found between rad-scores and WORMS subscores of cartilage (r = 0.29–0.54, all *p* < 0.01), bone (r = 0.43–0.56, all *p* < 0.01), meniscus (r = 0.20–0.30, all *p* ≤ 0.01), ligament (r = 0.47, *p* < 0.01), and synovium (r = 0.70, *p* < 0.01) (Table 3; Fig. 6).

**Table 3:** The correlation between radiomic signature and WORMS

	Total region		PF		MFT		LFT	
	r (95%CI)	p value	r (95%CI)	p value	r (95%CI)	p value	r (95%CI)	p value
Total structure	0.63 (0.52–0.72)	<0.01	0.46 (0.33–0.58)	<0.01	0.57 (0.47–0.68)	<0.01	0.45 (0.32–0.57)	<0.01
Cartilage	0.54 (0.41–0.64)	<0.01	0.44 (0.31–0.56)	<0.01	0.52 (0.41–0.63)	<0.01	0.29 (0.14 ~ 0.42)	<0.01
Bone	0.56 (0.44–0.67)	<0.01	0.46 (0.33–0.58)	<0.01	0.56 (0.45–0.66)	<0.01	0.43 (0.29–0.56)	<0.01
Meniscus	0.30 (0.16–0.44)	<0.01	-	-	0.30 (0.15–0.42)	<0.01	0.20 (0.06–0.35)	0.01
Ligaments	0.41 (0.27–0.53)	<0.01	-	-	-	-	-	-
Synovium	0.70 (0.62–0.77)	<0.01	-	-	-	-	-	-

Note. PF, patellofemoral joint; MFT, medial femorotibial joint; LFT, lateral femorotibial joint. “Total region” represents all regions of PF, MFT, and LFT; “Total structure” represents all structures of cartilage, bone, meniscus, ligaments, and synovium

## Discussion

IFP may play a beneficial or detrimental role in KOA depending on the biomechanical, anticatabolic, proinflammatory or metabolic properties. Therefore, evaluation of IFP can play a key role in the diagnostic and clinical management of KOA [3, 4, 6, 7, 9–13]. MRI parameters of signal intensity, shape, volume, perfusion and others have frequently been used to evaluate IFP alteration in patients with KOA [14–16, 18, 19]. However, these methods are of substantial variability, with side effects and high cost. In this study, we developed a novel radiomic signature to detect IFP abnormality in older adults with KOA. Our study showed the following findings: (1) KOA-related IFP abnormality can be evaluated by the rad-scores quantitatively and meaningfully; (2) the rad-scores were associated with the severity of KOA based on WORMS scoring; and (3) the rad-scores were significantly and positively associated with knee structural abnormality, especially synovium, bone and cartilage abnormality.

Pathological studies of KOA have reported that vascular neoformations, edema, fibrosis, and inflammatory reaction occur in the IFP, leading to an increase in the heterogeneity of IFP [3, 6, 7]. In this study, the AUCs of the radiomics signature in training and test dataset were greater than 0.75. The good performance of the radiomics signature suggested it may represent an accurate and effective means of evaluation to detect IFP heterogeneity in KOA. We delineated the whole IFP with more objective and comprehensive information compared with measurement of morphology [27] or lesion signal intensity deviation, including shape feature and first-order features, second-order features, and these features combined with filters [14, 19, 27, 28]. Our findings identified eight radiomic features, and the feature with the most weighted was wavelet-HLL_glszm_LargeAreaHighGrayLevelEmphasis, which measures the joint distribution of larger size zones with higher gray-level values in the wavelet-HLL images [28]. Seven of the eight features were extracted from wavelet filtered images, indicating that features of wavelet filters better reflect the biological characteristics and heterogeneity of IFP in KOA. Wavelet filter is a time-frequency analysis method that is beneficial to image sharpening and noise elimination [29]. Three of the eight features were firstorder_Kurtosis, which describes the difference in the grayscale intensity distribution [28].

Our results showed that the radiomic signature of IFP can discriminate patients with KOA and is positively and consistently associated with WORMS scoring of total knee structures. WORMS, a semiquantitative, multi-feature evaluation method of KOA in conventional MRI, provides a measurement of severity and potentially relevant structure abnormality of KOA; this method has been widely used in many clinical and epidemiological trials [26, 30]. Thus, the close correlation of the radiomic signature and WORMS scoring suggested that the severity of KOA could be reflected by the radiomic signature. A higher value of rad-score meant greater severity of KOA, and IFP may play an important role in the progression of KOA.

IFP radiomic alternation in KOA patients was significantly correlated with knee structural abnormalities, in which cartilage and bone abnormality had a higher absolute r value of the correlation coefficient. This result suggested that IFP is more closely related to cartilage and bone in KOA. The IFP secretes cytokines and adipokines such as leptin, adiponectin, IL-6, and fibroblast growth factor, which affect cartilage and participate in osteophyte formation [12, 13, 31, 32]. These joint structures can also adjust the IFP [3]. Previous studies have also shown that IFP alteration on MRI, such as signal intensity, maximum area, and fat fraction, was significantly correlated with knee structural abnormalities, but limited to cartilage defects, bone marrow lesions, and synovitis [14, 25, 27, 33]. In this study, the knee structure description was more comprehensive and also included bone cysts, bone attrition, osteophytes, meniscus, and ligaments.

This study has several limitations. First, it was a cross-sectional and retrospective study, without prospective research and longitudinal study. Whether the radiomic signature is a biomarker to predict the IFP development of KOA is unclear. However, our results indicate that the radiomic signature predicts IFP alteration of KOA, which provides a new perspective for future research. Second, this was a single-center study, and the size and category of samples were not considerable. Expanding the sample size and covering a larger population are necessary to represent cross-regional and ethnic groups in future research. Third, histological examinations were unable to performed in this study, so the relationship between radiomic signature and IFP histological abnormality remain unclear; these examinations should be pursued in future studies. Finally, our grouping design was based on the synthesis of previous literature reports and the matching of individual factors such as age, sex, and other physical factors of patients. Therefore, this study is of certain reliability and research significance.

## Conclusion

The radiomic signature established in this study may be a reliable biomarker to detect IFP abnormality of KOA. Radiomic alterations in IFP were associated with knee structural abnormalities, especially synovium, cartilage, and subchondral bone abnormality, in older adults.

## Electronic supplementary material

Below is the link to the electronic supplementary material.

Supplementary Material 1

## Data Availability

The datasets analyzed during the current study are available from the corresponding author on reasonable request.

## Abbreviations

AUC: Area under the curve
GLCM: Gray level cooccurrence matrix
GLDM: Gray level dependence matrix
GLRLM: Gray level run length matrix
GLSZM: Gray level size zone matrix
IFP: Infrapatellar fat pad
KL: Kellgren–Lawrence
KOA: Knee osteoarthritis
LR: Logistic regression
MRI: Magnetic resonance imaging
mRMR: Minimum redundancy maximum relevance
NGTDM: Neighboring gray tone difference matrix
ROC: Receiver operating characteristic
RSD: Relative standard deviation
SVM: Support vector machine
WORMS: Whole-organ magnetic resonance imaging score

## References

[1] Katz JN, Arant KR, Loeser RF (2021). Diagnosis and treatment of hip and knee osteoarthritis: a review. JAMA.

[2] Litwic A, Edwards MH, Dennison EM, Cooper C (2013). Epidemiology and burden of osteoarthritis. Br Med Bull.

[3] Zeng N, Yan ZP, Chen XY, Ni GX (2020). Infrapatellar Fat pad and knee osteoarthritis. Aging Dis.

[4] Poole AR (2012). Osteoarthritis as a whole Joint Disease. HSS J.

[5] Macchi V, Stocco E, Stecco C, Belluzzi E, Favero M, Porzionato A (2018). The infrapatellar fat pad and the synovial membrane: an anatomo-functional unit. J Anat.

[6] Favero M, El-Hadi H, Belluzzi E, Granzotto M, Porzionato A, Sarasin G (2017). Infrapatellar fat pad features in osteoarthritis: a histopathological and molecular study. Rheumatology.

[7] An JS, Tsuji K, Onuma H, Araya N, Isono M, Hoshino T (2021). Inhibition of fibrotic changes in infrapatellar fat pad alleviates persistent pain and articular cartilage degeneration in monoiodoacetic acid-induced rat arthritis model. Osteoarthr Cartil.

[8] Eymard F, Pigenet A, Citadelle D, Tordjman J, Foucher L, Rose C (2017). Knee and hip intra-articular adipose tissues (IAATs) compared with autologous subcutaneous adipose tissue: a specific phenotype for a central player in osteoarthritis. Ann Rheum Dis.

[9] Apinun J, Sengprasert P, Yuktanandana P, Ngarmukos S, Tanavalee A, Reantragoon R. Immune Mediators in Osteoarthritis: Infrapatellar Fat Pad-Infiltrating CD8 + T Cells Are Increased in Osteoarthritic Patients with Higher Clinical Radiographic Grading. International Journal of Rheumatology 2016, 2016:1–8. 10.1155/2016/9525724 PMC5192329 28070192

[10] Orlowsky EW, Kraus VB (2015). The role of Innate Immunity in Osteoarthritis: when our First line of Defense goes on the Offensive. J Rhuematol.

[11] Sae-jung T, Leearamwat N, Chaiseema N, Sengprasert P, Ngarmukos S, Yuktananda P (2021). The infrapatellar fat pad produces interleukin‐6‐secreting T cells in response to a proteoglycan aggrecan peptide and provides dominant soluble mediators different from that present in synovial fluid. Int J Rheum Dis.

[12] Hui W, Litherland GJ, Elias MS, Kitson GI, Cawston TE, Rowan AD (2012). Leptin produced by joint white adipose tissue induces cartilage degradation via upregulation and activation of matrix metalloproteinases. Ann Rheum Dis.

[13] He J, Jiang Y, Alexander PG, Ulici V, Zhu Y, Wu S (2017). Infrapatellar fat pad aggravates degeneration of acute traumatized cartilage: a possible role for interleukin-6. Osteoarthr Cartil.

[14] Han W, Aitken D, Zhu Z, Halliday A, Wang X, Antony B (2016). Signal intensity alteration in the infrapatellar fat pad at baseline for the prediction of knee symptoms and structure in older adults: a cohort study. Ann Rheum Dis.

[15] Wang K, Ding C, Hannon MJ, Chen Z, Kwoh CK, Hunter DJ (2018). Quantitative Signal Intensity Alteration in Infrapatellar Fat Pad Predicts Incident Radiographic Osteoarthritis: the Osteoarthritis Initiative. Arthritis Care Res.

[16] Lu M, Chen Z, Han W, Zhu Z, Jin X, Hunter DJ (2016). A novel method for assessing signal intensity within infrapatellar fat pad on MR images in patients with knee osteoarthritis. Osteoarthr Cartil.

[17] Cai J, Xu J, Wang K, Zheng S, He F, Huan S (2015). Association between Infrapatellar Fat Pad volume and knee structural changes in patients with knee osteoarthritis. J Rhuematol.

[18] van der Heijden RA, de Vries BA, Poot DHJ, van Middelkoop M, Bierma-Zeinstra SMA, Krestin GP (2021). Quantitative volume and dynamic contrast-enhanced MRI derived perfusion of the infrapatellar fat pad in patellofemoral pain. Quant Imaging Med Surg.

[19] de Vries BA, van der Heijden RA, Poot DHJ, van Middelkoop M, Meuffels DE, Krestin GP (2020). Quantitative DCE-MRI demonstrates increased blood perfusion in Hoffa’s fat pad signal abnormalities in knee osteoarthritis, but not in patellofemoral pain. Eur Radiol.

[20] Xie Y, Dan Y, Tao H, Wang C, Zhang C, Wang Y (2021). Radiomics feature analysis of cartilage and subchondral bone in differentiating knees predisposed to Posttraumatic Osteoarthritis after Anterior Cruciate Ligament Reconstruction from healthy knees. Biomed Res Int.

[21] Hirvasniemi J, Klein S, Bierma-Zeinstra S, Vernooij MW, Schiphof D, Oei EHG (2021). A machine learning approach to distinguish between knees without and with osteoarthritis using MRI-based radiomic features from tibial bone. Eur Radiol.

[22] MacKay JW, Murray PJ, Kasmai B, Johnson G, Donell ST, Toms AP (2017). Subchondral bone in osteoarthritis: association between MRI texture analysis and histomorphometry. Osteoarthr Cartil.

[23] Teoh YX, Lai KW, Usman J, Goh SL, Mohafez H, Hasikin K (2022). Discovering knee osteoarthritis imaging features for diagnosis and prognosis: review of Manual Imaging Grading and Machine Learning Approaches. J Healthc Eng.

[24] Collins NJ, Oei EHG, Kanter JL, Vicenzino B, Crossley KM (2019). Prevalence of Radiographic and magnetic resonance imaging features of Patellofemoral Osteoarthritis in Young and Middle-Aged adults with Persistent Patellofemoral Pain. Arthritis Care Res.

[25] Chen Y, Zhang X, Li M, Zhong L, Ding Y, Zhang Y (2022). Quantitative MR evaluation of the infrapatellar fat pad for knee osteoarthritis: using proton density fat fraction and T2* relaxation based on DIXON. Eur Radiol.

[26] Peterfy CG, Guermazi A, Zaim S, Tirman PFJ, Miaux Y, White D (2004). Whole-organ magnetic resonance imaging score (WORMS) of the knee in osteoarthritis. Osteoarthr Cartil.

[27] Han W, Cai S, Liu Z, Jin X, Wang X, Antony B (2014). Infrapatellar fat pad in the knee: is local fat good or bad for knee osteoarthritis?. Arthritis Res Therapy.

[28] van Griethuysen JJM, Fedorov A, Parmar C, Hosny A, Aucoin N, Narayan V (2017). Computational Radiomics System to Decode the Radiographic phenotype. Cancer Res.

[29] Qian Z, Li Y, Wang Y, Li L, Li R, Wang K (2019). Differentiation of glioblastoma from solitary brain metastases using radiomic machine-learning classifiers. Cancer Lett.

[30] Jungmann PM, Tham S-C, Liebl H, Nevitt MC, McCulloch CE, Lynch J (2013). Association of trochlear dysplasia with degenerative abnormalities in the knee: data from the Osteoarthritis Initiative. Skeletal Radiol.

[31] Clockaerts S, Bastiaansen-Jenniskens YM, Runhaar J, Van Osch GJVM, Van Offel JF, Verhaar JAN (2010). The infrapatellar fat pad should be considered as an active osteoarthritic joint tissue: a narrative review. Osteoarthr Cartil.

[32] Reinke L, Lam AP, Flozak AS, Varga J, Gottardi CJ (2016). Adiponectin inhibits wnt co-receptor, Lrp6, phosphorylation and β-catenin signaling. Biochem Biophys Res Commun.

[33] Zhong L, Li M, Du X, Ding Y, Zhang X, Mei Y (2022). Quantitative evaluation of the characteristic of infrapatellar fat pad Fat Content and Unsaturation Index by using hydrogen proton MR spectroscopy. Magn Reson Imaging.

