# Supplementary material for: Quantitative evaluation of the infrapatellar fat pad in knee osteoarthritis: MRI-based radiomic signature
Source: BMC Musculoskelet Disord. 2023 Apr 25;24:326. doi: 10.1186/s12891-023-06433-7 (PMC10127010; doi:10.1186/s12891-023-06433-7)
Supplement: Supplementary file 1 — Supplementary Material 1 [file 12891_2023_6433_MOESM1_ESM.doc]

**Supplement Material**

**Part I. Dimension reduction of stable radiomic features**

First, analysis of variance on the extracted features was performed to measure the dispersion of numeric data. A larger variance indicated a greater fluctuation of data. According to the condition that the features greater than the threshold 1 were retained, 419 radiomic features were left.

Second, we used the minimum redundancy maximum relevance (mRMR) algorithm to obtain optimal features with high correlation and low redundancy. The minimum redundancy algorithm calculated the paired correlation between two features, and features with correlation coefficients lower than 0.8 were left for low redundancy. The maximum relevance algorithm calculated the correlation between feature and knee osteoarthritis outcome; features with correlation coefficients greater than 0.1 were left for high correlation. A total of 24 radiomic features were retained.

Finally, the gradient boosting decision tree (GBDT) algorithm was used to classify or regress data by a linear combination of basis functions and reduce the residual generated in the training process. After applying GBDT for selecting features, there were eight radiomic features left.

**Part II. Selection of the machine learning algorithm**

The relative standard deviation (RSD) is the absolute value of the coefficient of variation and is often expressed as a percentage. The equation of RSD is: , where and are the standard deviation and mean of the 1000 AUC values from 1000 Bootstrap replication in the training set, respectively (Table S3). The RSD (mean±SD) of logistic regression (LR), Bayes, and support vector machine (SVM) was 5.74%, 6.57%, and 18.53%, respectively. Therefore, LR, with a minimal RSD value, was chosen.

**Part III. The KOA-related IFP radiomic signature by** **machine-learning algorithm of LR**

The KOA-related IFP radiomic signature was constructed and the radiomic score (Rad-score) was calculated. The equation was as follows:

Rad-score=1.7548+0.377*[original_glcm_Correlation]

+0.3137*[wavelet-LHL_firstorder_Kurtosis]

+0.9032*[wavelet-LHL_firstorder_Mean]

-0.1999*[wavelet-LHH_glszm_LargeAreaHighGrayLevelEmphasis]

-0.58*[wavelet-HLL_firstorder_Kurtosis]

+1.057*[wavelet-HLL_glszm_LargeAreaHighGrayLevelEmphasis]

+0.3301*[wavelet-HLH_ngtdm_Contrast]

-0.9676*[wavelet-HHL_firstorder_Kurtosis]

Table S1 The imaging protocol parameters for different sequences

| Sequence | Acquisition | TR  (ms) | TE  (ms) | FA  (deg) | Slice thickness  (mm) | FOV  (mm×mm×mm) | Matrix size | Voxel size  (mm×mm) | NSA |
| --- | --- | --- | --- | --- | --- | --- | --- | --- | --- |
| T1WI | Sagittal | 587 | 8 | 90 | 3.5 | 160×160×92 | 228×194 | 0.70×0.80 | 1 |
| PDWI | Sagittal | 2497 | 30 | 90 | 3.5 | 160×160×92 | 228×183 | 0.70×0.82 | 1 |
| PDWI | Coronal | 2661 | 30 | 90 | 3.5 | 160×160×84 | 228×180 | 0.70×0.80 | 2 |
| PDWI | Transversal | 2418 | 30 | 90 | 4 | 150×150×109 | 272×225 | 0.55×0.65 | 1.5 |

Note. TR, repetition time; TE, echo time; FA, flip angle; FOV, field of view; NSA, number of signal averages.

Table S2 The summary of the modified WORMS

|  |  | **PF WORMS** | | **MFT WORMS** | | **LFT WORMS** | | **Total structure score** |
| --- | --- | --- | --- | --- | --- | --- | --- | --- |
|  |  |  | Max score |  | Max score |  | Max score |
| **Cartilage WORMS** | |  |  |  |  |  |  |  |
| 0 | normal thickness and signal | Patella | 6 | Medial femur | 6 | Lateral femur | 6 | 36 |
| 1 | normal thickness but increased signal |
| 2 | partial-thickness defect <1 cm |
| 2.5 | full-thickness defect <1 cm |
| 3 | multiple areas of partial-thickness defect, or >1 cm but <75% of the region | Femur | 6 | Medial tibia | 6 | Lateral tibia | 6 |
| 4 | ≥75% of the region |
| 5 | multiple areas of full-thickness loss or a >1 cm but <75% of the region |
| 6 | ≥75% of the region |
| **Bone WORMS** | |  |  |  |  |  |  |  |
| Bone marrow abnormality | |  |  |  |  |  |  |  |
| 0 | none | Patella | 3 | Medial femur | 3 | Lateral femur | 3 | 18 |
| 1 | <25% of the region |
| 2 | 25%–50% of the region | Femur | 3 | Medial tibia | 3 | Lateral tibia | 3 |
| 3 | >50% of the region |
| Bone cysts | |  |  |  |  |  |  |  |
| 0 | none | Patella | 3 | Medial femur | 3 | Lateral femur | 3 | 18 |
| 1 | <25% of the region |
| 2 | 25%–50% of the region | Femur | 3 | Medial tibia | 3 | Lateral tibia | 3 |
| 3 | >50% of the region |
| Bone attrition | |  |  |  |  |  |  |  |
| 0 | normal | Patella | 3 | Medial femur | 3 | Lateral femur | 3 | 18 |
| 1 | flattening |
| 2 | slight concavity | Femur | 3 | Medial tibia | 3 | Lateral tibia | 3 |
| 3 | marked concavity |
| Osteophytes | |  |  |  |  |  |  |  |
| 0 | none | Patella | 7 | Medial femur | 7 | Lateral femur | 7 | 21 |
| 1 | equivocal |
| 2 | small |
| 3 | small-moderate |
| 4 | moderate | Femur | 7 | Medial tibia | 7 | Lateral tibia | 7 | 21 |
| 5 | moderate-large |
| 6 | large |
| 7 | very large |
| **Meniscus WORMS** six compartments (medial and lateral: anterior, body, posterior) | | | | | | | | |
| 0 | normal | － | － | Medial Meniscus | 6 | Lateral Meniscus | 6 | 12 |
| 1 | one or more compartments were intra-substance abnormalities |
| 2 | one compartment was non-displaced tear |
| 3 | more than one compartment was graded as non-displaced tear |
| 4 | one or more compartments were complex tear |
| 5 | one compartment was graded as maceration |
| 6 | more than one compartment was graded as maceration |
| **Ligaments WORMS** ACL scores + PCL scores + 1/2 (MCL scores and LCL scores) | | | | | | | | |
| 0 | intact | － | － | － | － | － | － | 3 |
| 1 | torn |
| **Synovium WORMS** | |  |  |  |  |  |  |  |
| effusion-synovitis | |  |  |  |  |  |  |  |
| 0 | normal | － | － | － | － | － | － | 3 |
| 1 | <33% of maximum potential distention |
| 2 | 33%–66% of maximum potential distention |
| 3 | >66% of maximum potential distention |
| Hoffa-synovitis | | － | － | － | － | － | － | 3 |
| 0 | normal |
| 1 | mild |
| 2 | moderate |
| 3 | severe |
| **Total region score** | | PF | 45 | MFT | 51 | LFT | 51 | 153 |

Note. WORMS, whole organ magnetic resonance imaging score; PF, patellofemoral joint; MFT, medial femorotibial joint; LFT, lateral femorotibial joint; ACL, anterior cruciate ligament; PCL, posterior cruciate ligament; MCL, medial collateral ligament; LCL, lateral collateral ligament.

Table S3. The specific data of three machine-learning algorithms

|  | AUC | Accuracy | Precision | Specificity | Sensitivity |
| --- | --- | --- | --- | --- | --- |
| LR, mean (SD) | 0.801(0.046) | 0.746(0.071) | 0.929(0.028) | 0.778(0.109) | 0.738(0.109) |
| Bayes, mean (SD) | 0.776(0.051) | 0.816(0.023) | 0.853(0.030) | 0.390(0.167) | 0.930(0.042) |
| SVM, mean (SD) | 0.788(0.146) | 0.789(0.167) | 0.780(0.212) | 0.232(0.269) | 0.989(0.023) |

Note. LR, logistic regression; SVM, support vector machine; SD, standard deviation.


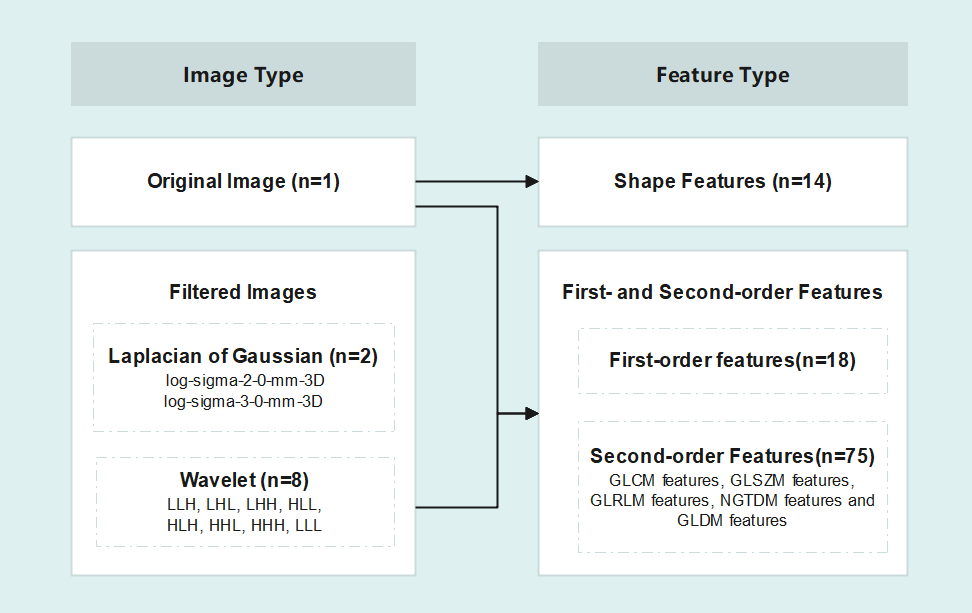


Figure S1 Summary of the extracted features.
